# Transcriptome analysis reveals the regulatory mode by which NAA promotes the growth of *Armillaria gallica*

**DOI:** 10.1371/journal.pone.0277701

**Published:** 2022-11-21

**Authors:** Jinlong Cai, Bilian Chen, Wenchao Li, Peng Xu, Yongguo Di, Huini Xu, Kunzhi Li

**Affiliations:** 1 Faculty of Life Science and Technology, Kunming University of Science and Technology, Kunming, China; 2 Planting Department of Zhaoyang District Bureau of Agriculture, Zhaotong, China; Nanjing Agricultural University, CHINA

## Abstract

A symbiotic relationship is observed between *Armillaria* and the Chinese herbal medicine *Gastrodia elata* (*G*. *elata*). *Armillaria* is a nutrient source for the growth of *G*. *elata*, and its nutrient metabolism efficiency affects the growth and development of *G*. *elata*. Auxin has been reported to stimulate *Armillaria* species, but the molecular mechanism remains unknown. We found that naphthalene acetic acid (NAA) can also promote the growth of *A*. *gallica*. Moreover, we identified a total of 2071 differentially expressed genes (DEGs) by analyzing the transcriptome sequencing data of *A*. *gallica* at 5 and 10 hour of NAA treatment. Gene Ontology (GO) and Kyoto Encyclopedia of Genes and Genomes (KEGG) analyses showed that these unigenes were significantly enriched in the metabolism pathways of arginine, proline, propanoate, phenylalanine and tryptophan. The expression levels of the general amino acid permease (Gap), ammonium transporter (AMT), glutamate dehydrogenase (GDH), glutamine synthetase (GS), Zn(II) 2Cys6 and C2H2 transcription factor genes were upregulated. Our transcriptome analysis showed that the amino acid and nitrogen metabolism pathways in *Armillaria* were rapidly induced within hours after NAA treatment. These results provide valuable insights into the molecular mechanisms by which NAA promotes the growth of *Armillaria* species.

## Introduction

*Gastrodia elata* (*G*. *elata*) is a valuable Chinese herbal medicine in China. This orchid species does not have roots and leaves, which can not absorb nutrients or perform photosynthesis. Thus, *G*. *elata* digests the symbiotic *Armillaria mellea* (*A*. *mellea*) to provide its nutrition [1–3]. *A*. *mellea* with a high growth rate can promote the growth of *G*. *elata*. Therefore, many studies have focused on promoting the growth of *A*. *mellea*. Screening tests for woody habitats suitable for the growth of *A*. *mellea* showed that white sandalwood, oak and walnut significantly promoted the growth of *A*. *mellea* while lilac inhibited the growth [4, 5]. Research on providing supplementary nutrition to *A*. *mellea* also showed that potato and carrot were beneficial to its growth [6]. Plant growth regulators, such as NAA [7], indole-3-acetic acid [8], 2,4-D [9], tannins [10], triacontanol and inositol [11], can regulate the growth of *A*. *mellea*. Promoting the growth of *A*. *mellea* has become an important field of *G*. *elata* research.

Fungi can provide plants with mineral nutrients. Arbuscular mycorrhiza (AM) represents a symbiotic association between a fungus (*Glomeromycota* spp.) and the roots of plant species [12–14]. The establishment of symbiosis relies on nutrient exchange between mycorrhizal fungi and plants. Nitrogen and phosphorus are the basic mineral nutrients for plant growth, and certain fungal species can promote plant uptake of mineral elements in soil, such as nitrogen and phosphorus [15, 16], and help plants absorb water and other trace mineral elements. The nutrient exchange between AM fungi and plants is the basis for maintaining their symbiotic relationship. Plants provide AM fungi with a carbon source for their growth [17–19], and in exchange, AM fungi provide plants with mineral nutrients, mainly phosphorus and nitrogen [20, 21]. Transporters in mycelia can absorb nitrogen nutrients, such as inorganic nitrogen and amino acids. At present, AMT genes from different mycorrhizal fungi have been isolated, and these transporter genes include *AMT1*, *AMT2* and *AMT3*, which belong to the *Mep*/*Amt* gene family [22, 23]. In yeast [24] and filamentous fungi [25, 26], amino acid transporters have been extensively and deeply studied. Recently, Cappellazzo et al. [27] isolated the amino acid transporter gene *Gmos AAP1* from AM fungi. Extraroot hyphae take up NH_4_^+^ and NO_3_^-^ from soil through AMT and nitrate transporters, respectively [28]. In mycelia, AM fungi reduce NO_3_^−^ to NH_4_^+^ through reductase. Then, glutamine synthetase synthesizes NH_4_^+^ and glutamic acid provided by plants into glutamine [29]. Through a series of biosyntheses, glutamine is converted into arginine [30], which is transported within the mycelium. Finally, arginine is degraded into NH_4_^+^ by the urea cycle, and NH_4_^+^ is then transferred into the exosome of the plant cytoplasmic membrane by a nitrogen transport ion pump and absorbed by the plant [31].

Although the establishment of symbiosis relies on nutrient exchange between mycorrhizal fungi and plants, the mechanism underlying arbuscular mycorrhiza formation remains poorly understood. Recent reports suggested that certain plant hormones are also important for arbuscule development. Abscisic acid, gibberellin acid and strigolactones have an important function in arbuscule maintenance and formation [32–35]. Although the mechanism of auxin in this type of plant–microbe interaction is unclear, studies have shown that auxin also plays a role in AM symbiosis [36–38]. The content of auxin varies in different mycorrhizal roots, with its content remaining stable in tobacco and leek mycorrhiza [39, 40] but increasing in mycorrhizal maize and soybean roots [41–43]. In the mycorrhizal roots of mutant nark soybeans with defects in the automatic regulation of nodulation, the increase in IAA content was low, indicating that IAA may have a function in the automatic regulation of mycorrhization [42]. Recent studies have found that auxin could play a role in AM colonization [36, 44]. Auxin perception and/or auxin signaling are important for arbuscule development [45, 46]. When cultured on medium supplemented with auxin, *A*. *mellea* grew vigorously and produced abundant rhizomorphs [47]. In culture, 2,4-dichlorophenoxyacetic acid and NAA could stimulate the growth rate of rhizomorphs of *A*. *mellea* [7, 9, 48]. Although NAA can promote the growth of *Armillaria*, its mechanism remains unclear.

In this study, we used RNA-seq analysis to determine the mechanism by which NAA promotes growth. Based on a DEG analysis, we proposed a hypothetical regulatory network of NAA that promotes the growth of *A*. *gallica*. In *A*. *gallica*, NAA promoted the expression of transcription factors, which in turn upregulated the expression levels of AMT and Gap genes. Then, the transcription levels of glutamate dehydrogenase and glutamine synthase genes were increased to promote amino acid and nitrogen metabolism in *A*. *gallica*, thereby promoting its growth. This hypothetical regulatory network might provide a theoretical basis for further studies on the molecular mechanism by which NAA promotes the growth of *A*. *gallica*.

## Materials and methods

### Culture of *A*. *gallica*

*A*. *gallica* strain AG01 was isolated from *G*. *elata* f. glauca in Zhao tong. To assess the ability of NAA to promote the growth of *A*. *gallica*, fungal cultures were prepared by adding 50 mL of melted (60°C) semisolid PDA (for 1 L PDA: 200 g peeled potatoes, 20 g dextrose, 2 g agar) medium to sterile tissue culture bottles containing NAA (8 mg/L) or solvent control. The inoculum used to seed the medium was a 0.5 cm tip of the rhizomorph. The fungi were incubated in the dark at 25°C. The rhizomorphs were separated from the media, and the dry weights were determined after 6, 12 and 18 days of growth. The results are based on one representative of three independent experiments.

Cultivated material was used to study the early response mechanism of *A*. *gallica* to NAA. The strain of *A*. *gallica* was inoculated in inclined tubes filled with PDA medium and cultured at 25°C for 10 days. When the mycelium had filled the inclined plane, it was transferred to liquid culture medium [49]. Then, the culture was shaken for 7 to 10 days at 115 rpm and 25°C. Based on previous experiments (S1 Fig), mycelium was collected immediately after 5 h and 10 h of NAA treatment, frozen in liquid nitrogen and stored at −80°C for further analysis.

### RNA extraction, library construction, and transcriptome sequencing

Total RNA was extracted with the RNAprep Pure Plant Kit (TIANGEN, China) and then quantified using a Nanodrop2000 spectrophotometer (NanoDrop Technologies, USA) and an Agilent 2100 Bioanalyzer (Agilent, USA).

For Illumina RNA sequencing, sequencing libraries were generated from the total RNA samples with the NEBNext® Ultra™ II RNA Library Prep Kit for Illumina (NEB, USA) following the manufacturer’s recommendations. The cDNA libraries were sequenced on an Illumina HiSeq 2000 platform (Illumina, USA).

### Quantification of gene expression levels

Gene expression levels were estimated by RSEM [50] for each sample as follows:

1. Clean data were mapped back onto the assembled transcriptome;
2. Read counts for each gene were obtained from the mapping results.

### Differential expression analysis

Differential expression analyses of pairs of groups were performed using the DESeq R package (1.10.1). The P values were adjusted using Benjamini and Hochberg’s approach for controlling the false discovery rate. Genes with an adjusted P value <0.05 found by DESeq were considered differentially expressed.

### GO and KEGG enrichment analysis of DEGs

Gene Ontology (GO) enrichment analyses of the differentially expressed genes (DEGs) were implemented by the top GO R package-based Kolmogorov–Smirnov test. We used KOBAS software [51] to test the statistical enrichment of differentially expressed genes in KEGG pathways.

### Quantitative PCR analysis

Total RNA was extracted from mycelium using RNAiso Plus (TaKaRa) according to the manufacturer’s recommended protocols. One microgram of total RNA was reverse-transcribed into first-strand cDNA with oligo dT primers using HiScript® II Reverse Transcriptase for RT–qPCR (Vazyme) following the manufacturer’s instructions, and cDNA templates were stored at −20°C until use. The RT–qPCR protocol consisted of an initial heat activation step of 95°C for 30 s, followed by 40 cycles of 95°C for 10 s and 60°C for 30 s. Three biological replicates were performed for each treatment, and each biological replicate consisted of three technical replicates.

The relative expression levels of the target genes were calculated using the 2^−ΔΔCT^ approach, with normalization of data to the geometric average of two reference control genes [52].

### Statistical analysis

Statistical analyses were performed using SPSS statistic 22.0 software. Duncan’s multiple test (*P* <0.05) was chosen for statistical analysis. Data are the means ± SE from at least three independent biological replicates.

## Results

### NAA promoted the growth of *A*. *gallica*

**Fig 1:**
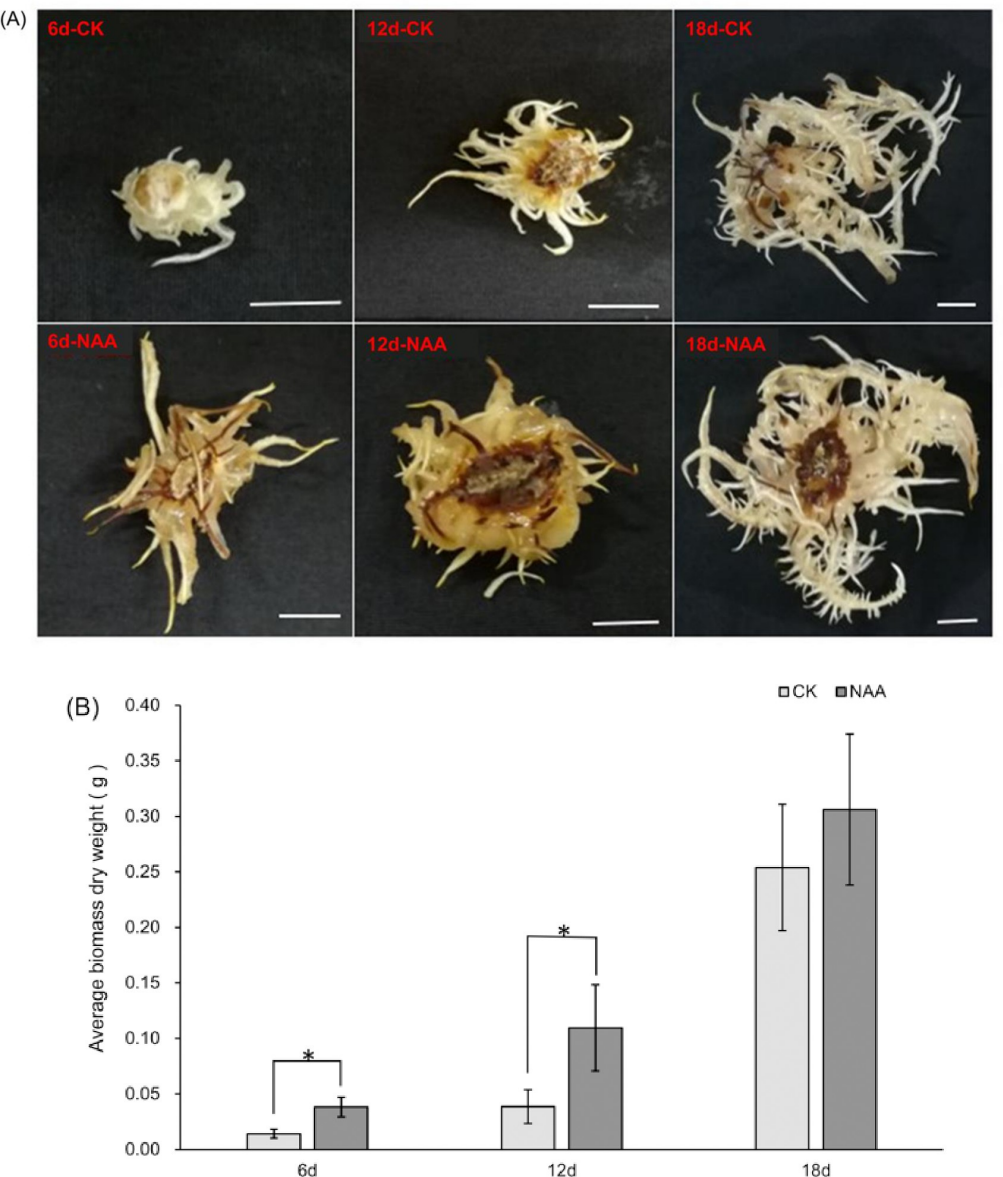
Effect of NAA on the growth of *A*. *gallica*. **(A)** Morphology of *A*. *gallica* after 6, 12 and 18 days of growth. *A*. *gallica* were cultured on medium with NAA or without NAA (CK). Scale bar = 1 cm. **(B)** Dry weights of *A*. *gallica* in different culture times. The values are the means ± SE of three biological replicates. Asterisks indicate significant differences (* p < 0.05, ANOVA).

We used 8 mg/L NAA to assess the effect of auxin treatment on the growth of *A*. *gallica*. On the medium with NAA, *A*. *gallica* grew profusely and consisted of many rhizomorphs (Fig 1A). At this NAA concentration, the dry weight of mycelium was significantly higher than that of the control after 6 and 12 days of *A*. *gallica* growth. However, significant differences from that of the control were not observed after 18 days of *A*. *gallica* growth (Fig 1B).

### Transcriptomic analysis of *A*. *gallica* mycelium in response to NAA at different time points

**Fig 2:**
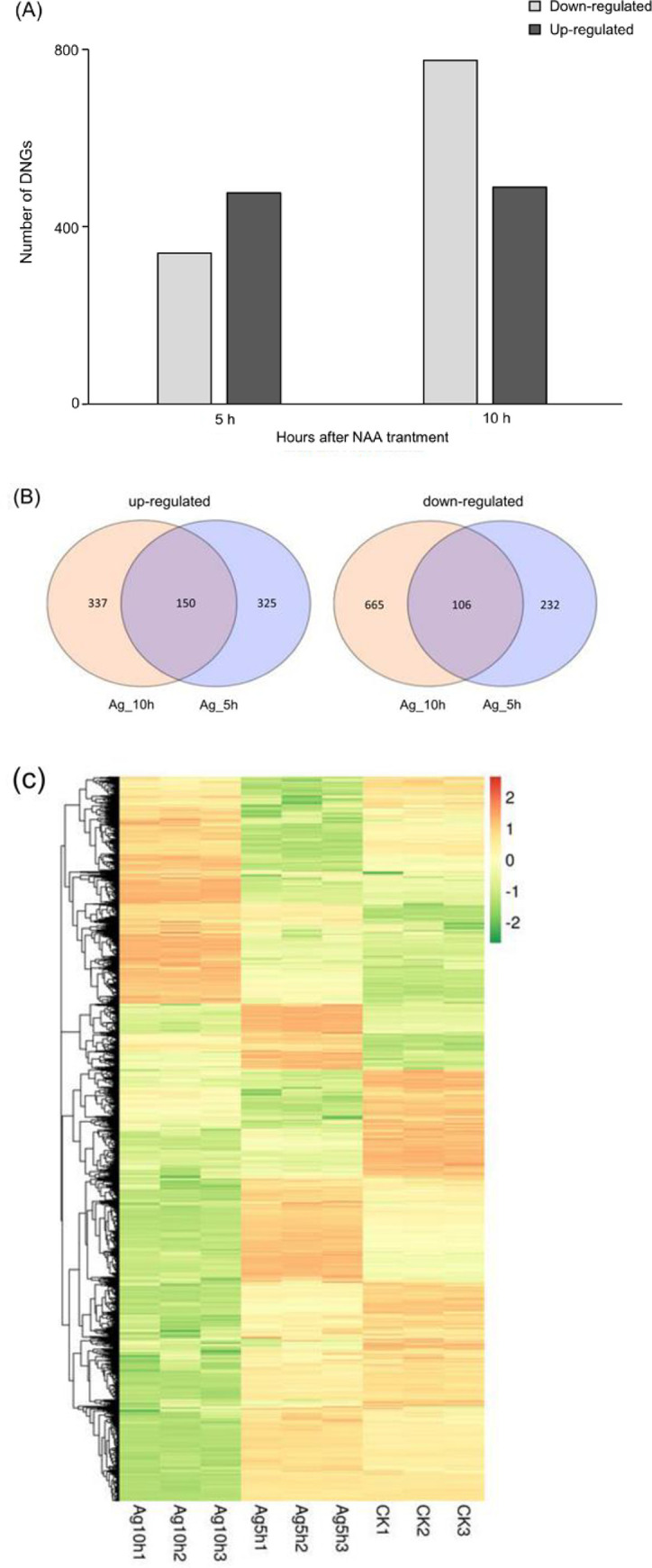
Transcriptomic changes in *A*. *gallica* in response to NAA treatment at 5 and 10 h. **(A)** Numbers of DEGs of the treatments; **(B)** Venn diagram illustrating the number of DEGs among the treatments; **(C)** Heatmap showing the relative expression levels of DEGs under NAA treatment.

To analyze the genes that may participate in NAA-promoted *A*. *gallica* mycelium growth, differences in gene expression were examined in the mycelium of *A*. *gallica* treated with NAA for 5 and 10 h. A total of 2071 DEGs were identified in *A*. *gallica* mycelium, 813 at 5 h and 1258 at 10 h, respectively (Fig 2A). The distribution of up- and downregulated genes was counted for each time point and is shown in a Venn diagram (Fig 2B). After treatment with NAA for 5 and 10 h, a group of unique genes were upregulated (total 812), with 150 genes significantly upregulated at both time points. In addition, a number of genes were significantly downregulated (total 1003), with 106 genes showing reduced expression at both time points.

Furthermore, a heatmap (Fig 2C) was generated to provide an overview of the gene expression changes and expression pattern. The expression patterns of most DEGs subjected to the NAA treatment showed opposite trends at 5 h and 10 h (Fig 2C). Most of the genes with lower expression levels at 5 h had higher expression levels at 10 h, and vice versa. Compared with the CK (without NAA), the expression profiles of most DEGs under the NAA treatments showed great differences.

### Functional classification of the DEGs by GO and KEGG pathway analysis

**Fig 3:**
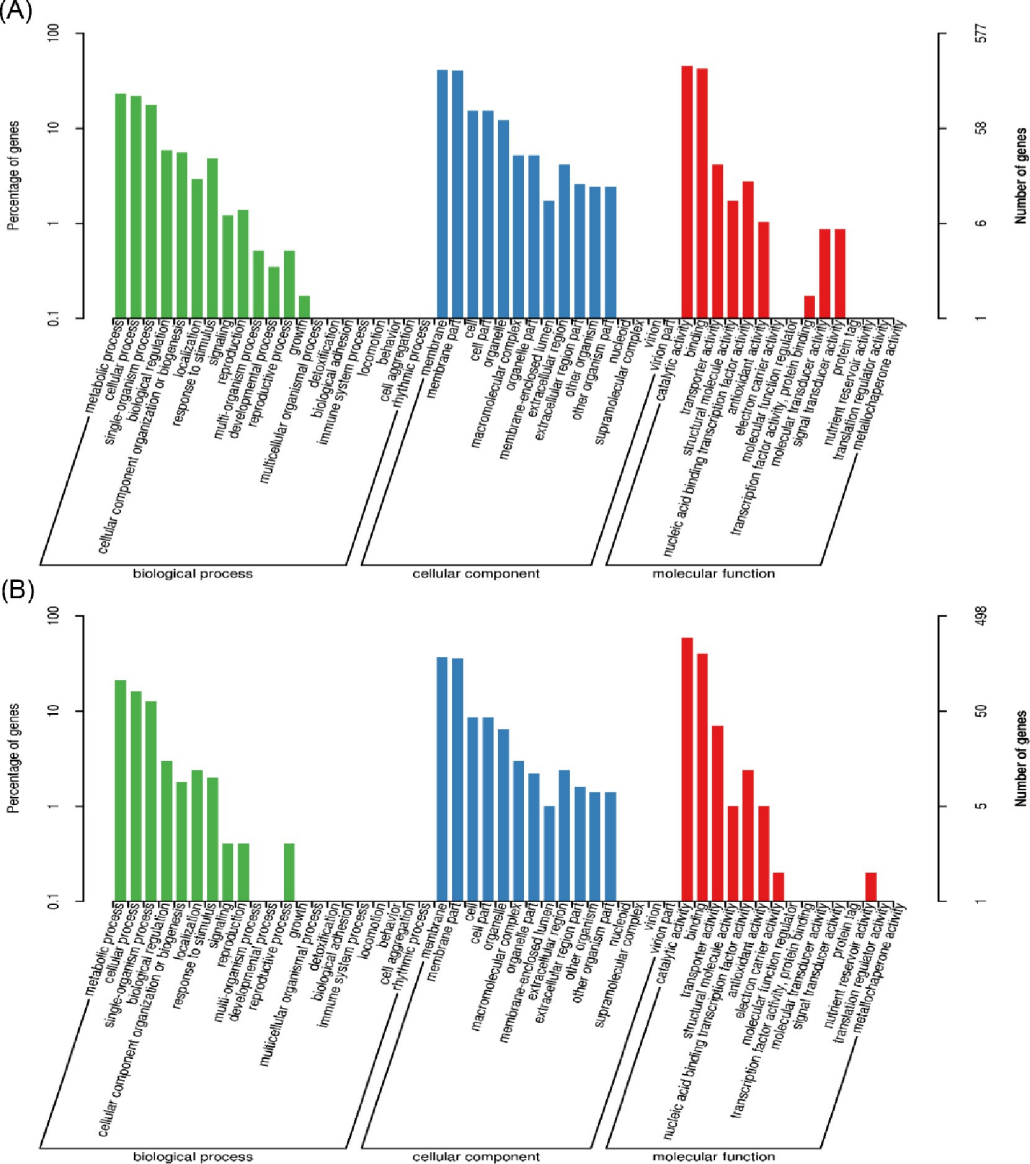
GO analysis of DEGs in *A*. *gallica* in response to NAA treatment at 5 and 10 h. **(A)** GO enrichment analysis of downregulated DEGs; **(B)** GO enrichment analysis of upregulated DEGs.

To identify NAA-induced genes, GO and KEGG pathway analyses were used to functionally classify the DEGs. In the GO analysis, the DEGs induced by NAA were classified into three main GO categories (Fig 3). At 5 h and 10 h (Fig 3), the DEGs in the biological process category were significantly enriched in metabolic processes, cellular processes and single-organism processes. The majority of DEGs in the molecular function category were enriched in catalytic activity and binding, although differences in molecular functions were observed. Only downregulated DEGs were enriched in molecular transducer activity and signal transducer activity, while only upregulated DEGs were also enriched in nutrient reservoir activity. The GO pathway analyses indicated that NAA promotes the metabolism of certain nutrients but inhibits the metabolism of others by acting on signaling pathways.

**Fig 4:**
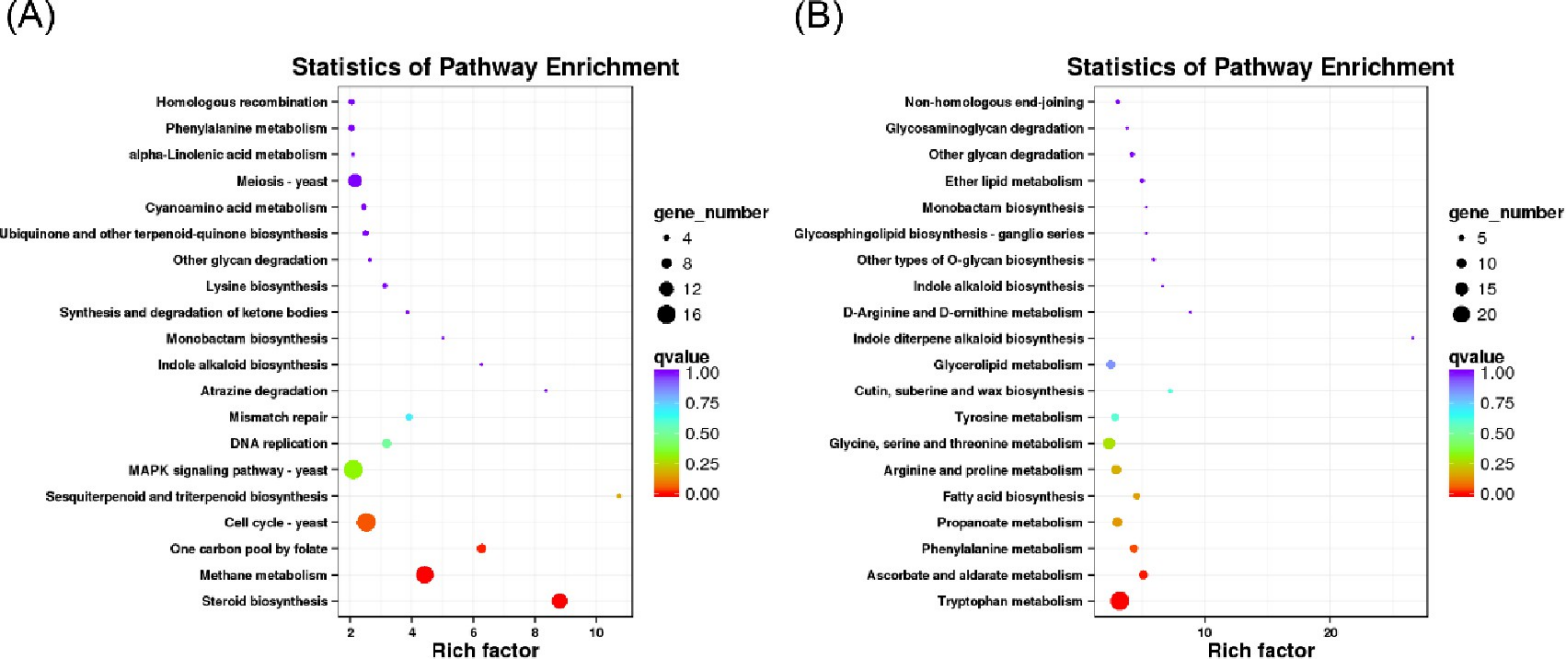
KEGG pathway enrichment analysis of DEGs. **(A)** KEGG enrichment analysis of downregulated DEGs; **(B)** KEGG enrichment analysis of upregulated DEGs. The Y-axis indicates the KEGG pathway, and the X-axis indicates the enrichment factor. The dot size represents the number of DEGs of the pathway, and the dot color indicates the q value.

To further reveal the biological function of NAA-induced DEGs, we performed enrichment analyses based on the KEGG database. The top 20 pathways for the most prominent DEGs were identified (Fig 4). The downregulated DEGs were mostly enriched in “sesquiterpenoid and triterpenoid biosynthesis”, “cell cycle-yeast”, “one carbon pool by folate”, “methane metabolism”, “steroid biosynthesis” and “tryptophan metabolism” (Fig 4A), while the upregulated DEGs were significantly enriched in “arginine and proline metabolism”, “fatty acid biosynthesis”, “propanoate metabolism”, “phenylalanine metabolism”, and “ascorbate and aldarate metabolism”. In particular, “tryptophan metabolism” was significantly enriched in the KEGG pathway analysis (Fig 4B). Transcriptome analysis showed that NAA positively affected amino acid metabolism in *A*. *gallica* and the transcript levels of nitrogen metabolism-associated genes.

In our current study, the transcript levels of 34 amino acid metabolism- and 4 nitrogen metabolism-related structural genes were analyzed, and the expression levels of those genes (except the nitrite reductase gene) were upregulated under NAA treatment (Table 1). Thirty-four putative ATM (amino acid transport and metabolism) genes were identified, among which the expression levels of 14 putative ATM genes were upregulated with NAA treatment at both time points. The transcript levels of 12 putative ATM genes were upregulated (1.02- to 1.38-fold) with NAA treatment at 10 h. Additionally, the other putative ATM genes in *A*. *gallica* were significantly induced (1.18- to 4.89-fold) in response to NAA treatment at both time points (Table 1). AMT, GS and GDH genes, which play an important role in nitrogen metabolism, were identified, and they displayed similar expression patterns under NAA treatment, with upregulation at 10 h, including c19578.graph_c0, c21900.graph_c0 and c24003.graph_c0.

**Table 1:** DEGs associated with amino acid and nitrogen metabolism in *A*. *gallica* in response to NAA treatment.

Gene Description	Gene ID	Nr_annotation	Log2Fold Change	
			5 h	10 h
Zn(Ⅱ)2Cys6 transcription factor	c26769.graph_c0	Hypothetical protein ARMGADRAFT_955812	1.04	/
	c18863.graph_c0	hypothetical protein ARMSODRAFT_1090953	1.02	1.73
	c24787.graph_c0	uncharacterized protein ARMOST_00211	1.08	/
	c27966.graph_c0	hypothetical protein ARMGADRAFT_638842	1.21	/
	c23323.graph_c0	hypothetical protein ARMSODRAFT_223645	1.15	/
	c15606.graph_c0	hypothetical protein ARMGADRAFT_1063831	1.03	/
	c24507.graph_c0	TPT-domain-containing protein	1.42	/
	c23156.graph_c0	hypothetical protein ARMGADRAFT_1071141	1.23	/
C2H2 Zin finger proteins	c26198.graph_c0	STE-domain-containing protein	1.06	/
	c24508.graph_c1	hypothetical protein ARMGADRAFT_1010957	1.16	/
	c24448.graph_c0	hypothetical protein ARMGADRAFT_1074413	/	1.33
	c27199.graph_c0	uncharacterized protein ARMOST_04702	/	1.53
	c10985.graph_c0	hypothetical protein ARMGADRAFT_1014537	1.11	/
	c16248.graph_c0	hypothetical protein ARMGADRAFT_997381	/	2.00
	c9941.graph_c0	hypothetical protein ARMGADRAFT_1065654	/	1.91
Amino acid transport and metabolism	c26398.graph_c0	high affinity methionine permease	1.44	1.89
	c23593.graph_c0	hypothetical protein ARMGADRAFT_1058748	1.23	/
	c19166.graph_c0	vacuolar amino acid permease	1.15	/
	c23409.graph_c0	related to uracil permease	1.36	/
	c26755.graph_c0	amino-acid permease inda1	/	1.27
	c19079.graph_c0	hypothetical protein ARMGADRAFT_1171443	/	1.2
	c26291.graph_c0	MFS general substrate transporter	/	1.02
	c25774.graph_c0	hypothetical protein ARMGADRAFT_1078564	/	1.36
	c26100.graph_c0	DAO-domain-containing protein	1.02	/
	c20438.graph_c0	PLP-dependent transferase	1.65	/
	c10068.graph_c0	hypothetical protein ARMGADRAFT_1004296	1.42	2.06
	c17479.graph_c0	hypothetical protein ARMGADRAFT_1036273	1.09	/
	c22013.graph_c0	aryl-alcohol oxidase precursor	1.98	/
	c22406.graph_c0	PLP-dependent transferase	/	1.38
	c23177.graph_c0	Clavaminate synthase-like protein	/	1.07
	c23191.graph_c0	Dehydrogenase ARMGADRAFT_1018426	2.05	/
	c23517.graph_c0	aryl-alcohol oxidase-like protein	1.20	/
	c23882.graph_c0	alcohol oxidase	1.52	1.94
	c23917.graph_c0	alpha/beta-hydrolase	/	1.32
	c24128.graph_c0	Homocysteine S-methyltransferase	/	1.10
	c24915.graph_c0	aryl-alcohol oxidase precursor	2.94	1.70
	c25006.graph_c1	pyranose dehydrogenase	/	1.06
	c25006.graph_c2	pyranose dehydrogenase	/	1.31
	c25251.graph_c0	related to Tyrosinase	1.03	/
	c25695.graph_c0	hypothetical protein ARMGADRAFT_735571	1.04	1.57
	c26168.graph_c0	NPD-domain-containing protein	1.18	/
	c26688.graph_c0	MATE efflux family protein	/	1.35
	c26823.graph_c0	glutaryl-CoA dehydrogenase	1.30	1.18
	c27283.graph_c0	hypothetical protein ARMGADRAFT_1033881	1.98	4.89
	c9872.graph_c0	acetylornithine aminotransferase, partial	/	1.31
	c27161.graph_c1	uncharacterized protein ARMOST_19843	1.21	/
	c27349.graph_c0	alcohol oxidase	2.73	1.41
	c27955.graph_c0	hypothetical protein ARMSODRAFT_942066	1.49	/
	c28019.graph_c0	aryl-alcohol oxidase precursor	1.89	/
Nitrite reductase	c17687.graph_c0	FAD/NAD(P)-binding domain-containing protein	/	-1.74
Ammonium transporter	c19578.graph_c0	ammonium transporter	/	1.93
Glutamine synthetase	c21900.graph_c0	related to RPL3-60s ribosomal protein l3	/	1.00
Glutamate dehydrogenase	c24003.graph_c0	NADP-specific glutamate dehydrogenase	/	1.42

“/” indicates no significant differences between the NAA treatment groups and CK.

### Expression profiling of transcription factors associated with amino acid metabolism and nitrogen metabolism

Transcription factors were significantly induced in response to NAA treatment, and they might play important regulatory roles in amino acid metabolism and nitrogen metabolism. In this study, a total of 21 transcription factors were identified as putative regulators of amino acid metabolism and nitrogen metabolism in response to NAA treatment. They included Zn(Ⅱ)_2_Cys_6_ (Zn2 Cys6 Zn clusters) and C2H2s (C2H2 zinc-finger proteins) (Table 1). The C2H2s might be the main determinant of amino acid and nitrogen metabolism in response to NAA in *A*. *gallica* because C2H2s accounted for the largest percentage. All 9 of the putative Zn(Ⅱ)_2_Cys_6_ TFs were upregulated under NAA treatment at 5 h. Among them, seven genes showed no change at 10 h while the other two genes were still significantly upregulated (Table 1). In this study, 12 putative C2H2 genes were identified, and most were upregulated under NAA treatment (Table 1). Furthermore, the expression levels of 7 putative C2H2s increased under NAA treatment at 5 h and the other 5 putative C2H2 genes showed upregulation at 10 h. These findings suggest that the above transcription factors might be involved in regulating NAA-promoted amino acid and nitrogen metabolism.

**Fig 5:**
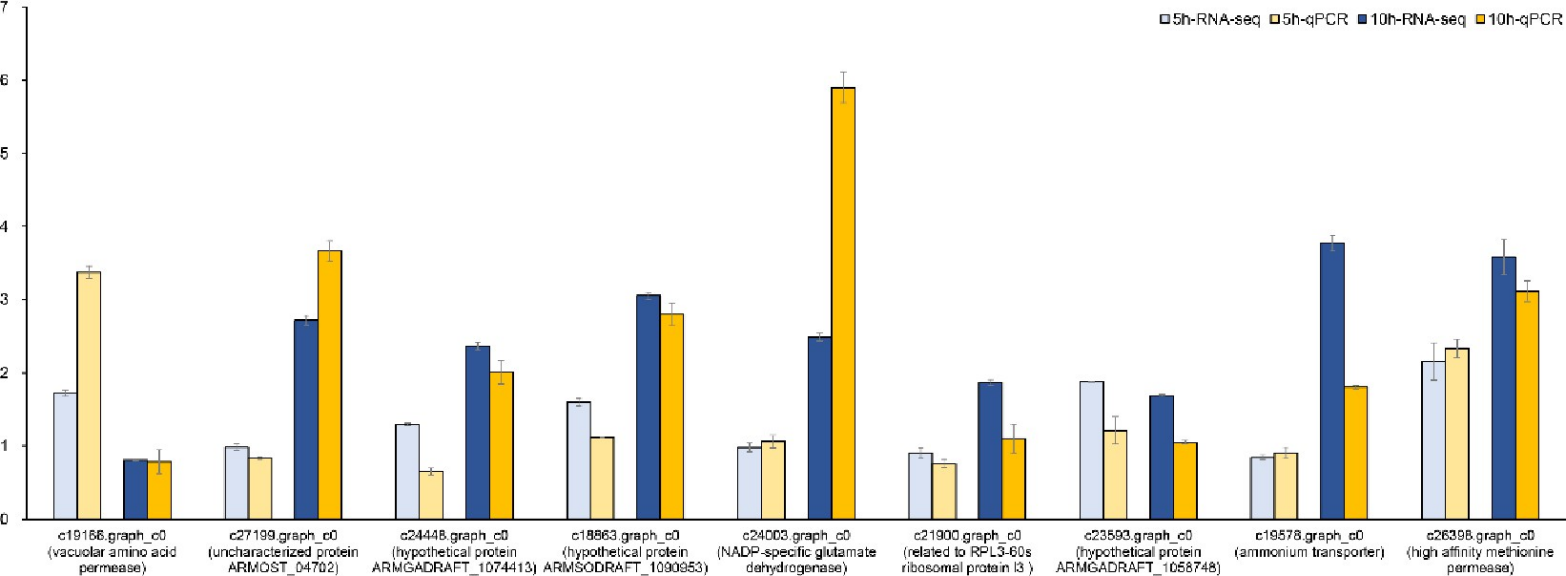
Expression of amino acid and nitrogen metabolism-related genes quantified by RNA-seq and qRT–PCR analyses. The y-axis represents the log2 FPKM values of genes from RNA-seq data and relative gene expression levels analyzed by qRT–PCR. Error bars mean the Standard error for three replicates.

qRT–PCR validation of differentially expressed genes was performed to validate whether the RNA-seq data truly reflected the actual transcription level. We selected 9 genes for quantitative real-time PCR (polymerase chain reaction) to detect DEG expression levels at 5 and 10 h (Fig 5). These genes included transcription factor genes and amino acid and nitrogen metabolism genes. In the qRT–PCR analysis, the expression patterns of these genes were very similar to the FPKM values from sequencing under the corresponding treatment, indicating that the RNA-seq data are reliable.

## Discussion

A symbiotic relationship is observed between *A*. *gallica* and *G*. *elata*, which is a traditional Chinese medicinal plant that obtains the nutrients needed for growth and development from *A*. *gallica*, which obtains nutrients by decomposing wood. To protect the environment and improve the yield of *G*. *elata*, the utilization efficiency of nutrients by *A*. *gallica* must be improved. A number of secondary metabolites play an important role in plant nutrient acquisition and abiotic stress tolerance. For example, under iron deficiency conditions, *Arabidopsis* relies on coumarin secretion to change the root microbial community [53]. The bacterial root microbiota, which is stimulated by secreted coumarins, promotes adaptions to iron-limited soil conditions [54]. IAA is involved in the establishment of biotrophy in *Piriformospora indica*-barley symbiosis [55]. IAA can promote fungal invasion and AM formation, especially at early stages [56]. Our findings were consistent with previous reports showing that NAA can also promote the growth and biomass of *Armillaria* [7, 47, 57]. Under NAA treatment, the dry weight of mycelium was significantly higher than that of the control after 6 and 12 days of growth (Fig 1). However, significant differences were not observed after 18 days (Fig 1B), which may be due to the continuous consumption of NAA as *A*. *gallica* grows until an ineffective concentration was reached at 18 days [7, 9, 48]. Auxin signal components have been suggested to be important nitrogen (N)-responsive regulators of root architecture. For example, in response to external N, mutants lacking ARF8 or AFB3 showed compromised root development [58, 59]. In *Arabidopsis*, auxin can be transported by the dual-affinity NO_3_^-^ transporter NRT1.1 [60] and plays a major role in lateral root emergence and growth induced by low N availability [61]. A recent study showed that the accumulation of auxin enhanced NO_3_^-^ uptake and assimilation. Auxin response factors promote N-use efficiency and grain yield by transactivating the expression of genes related to NO_3_^-^ metabolism [62]. These papers proved that auxin has beneficial biological effects in the nitrogen response. In the current study, we discovered that the expression of most genes associated with amino acid and nitrogen metabolism was upregulated by NAA (Table 1), including the genes encoding Gap, AMT, GDH and GS. However, the expression of the nitrite reductase gene was downregulated at 10 h, which might be related to the lack of nitrate nitrogen in the medium [49]. This study expands upon the knowledge of the molecular mechanisms underlying the ability of NAA to promote the growth of *Armillaria*.

NAA may promote nitrogen use efficiency and *Armillaria* growth by activating the expression of genes related to nitrogen and amino acid metabolism. Auxin response factors increase nitrogen use efficiency by promoting the expression of genes related to NO_3_^-^ metabolism [62]. Similarly, we found that NAA significantly promoted the expression levels of genes related to amino acid and nitrogen metabolism pathways. Moreover, the expression levels of 34 putative genes of ATM were upregulated under NAA treatment. With increasing NAA treatment time, the expression levels of *AMT*, *GS* and *GDH*, which play an important role in nitrogen metabolism, were all upregulated. AM fungi can take up NH_4_^+^ in soil through AMT [28] and then synthesize glutamine from NH_4_^+^ and glutamic acid through glutamine synthetase [29]. In this study, the expression levels of GHS and GS genes were increased, which may be related to the ability of AMT and ATM to transport NH_4_^+^ and amino acids into cells, respectively, resulting in increased NH_4_^+^ and amino acid contents in cells, which in turn promote the expression of *GS* and *GHS*.

Many researchers have extensively characterized transcription factors that regulate the expression of AMT and Gap genes in fungi. The transcription of the genes encoding ammonium permease and Gap was shown to be dependent on the transcription factor Gln3 in *Candida glabrata* [63]. Under low ammonium conditions, the deletion of the AREA transcription factor led to a significant reduction in the expression of the three predicted ammonium permease genes [64]. In rice, auxin-mediated promotion of NO_3_^-^ uptake is controlled by members of the OsARF family, such as OsARF6 and OsARF17, which synergistically promote NO_3_^-^ metabolism [62]. In this study, 21 transcription factors that responded to the NAA treatment were identified as putative regulators of amino acid and nitrogen metabolism, including Zn(II)2Cys6-encoding genes and C2H2-encoding genes (Table 1). Not only did the expression level of amino acid and nitrogen metabolism-related genes change with NAA treatment time, but the TF genes also had variable expression levels. This finding suggests that these TFs are likely the main regulators of amino acid and nitrogen metabolism-related genes in *A*. *gallica* under NAA treatment.

**Fig 6:**
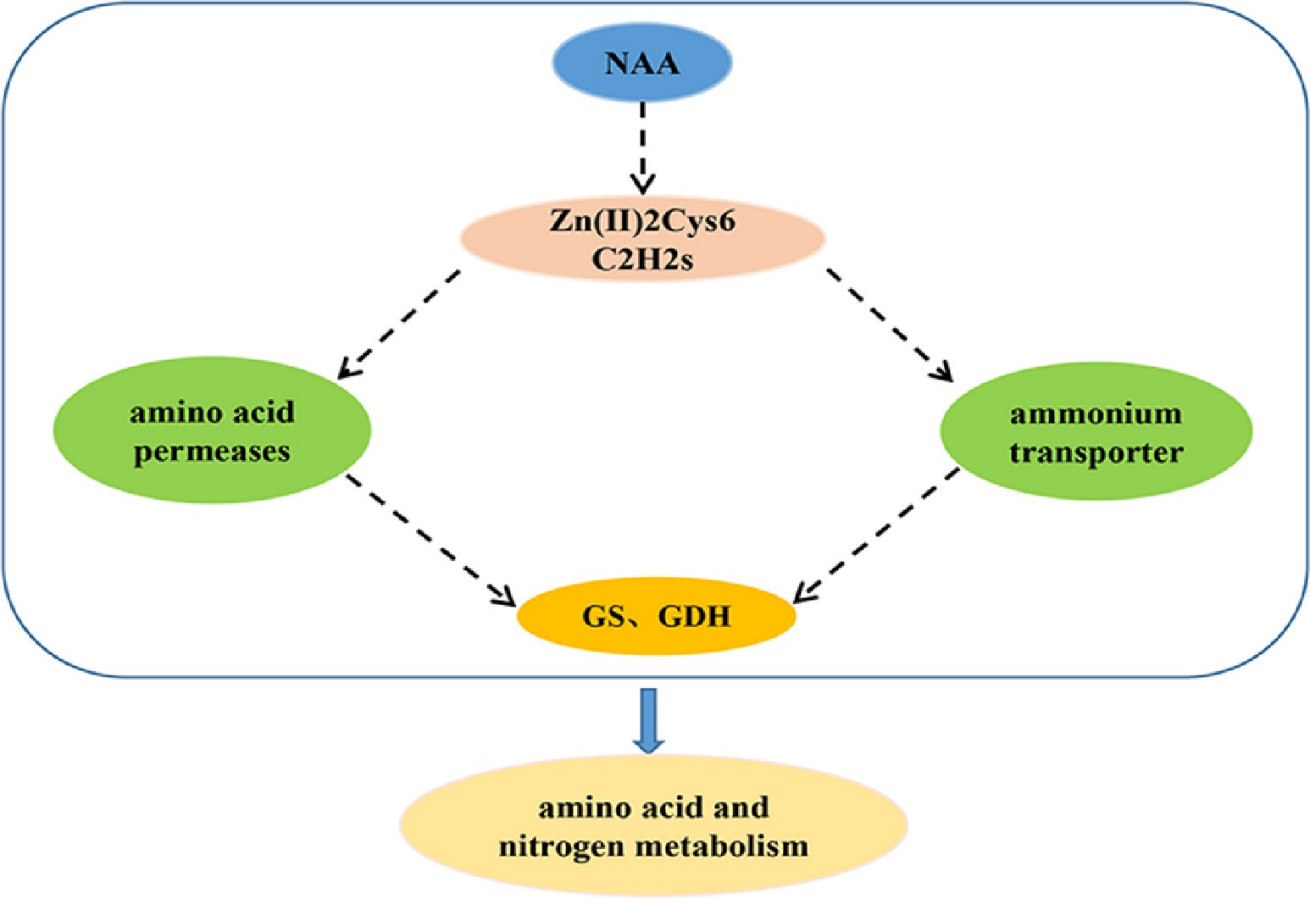
Speculative model of NAA promoting amino acid and nitrogen metabolism in *A*. *gallica*. Under NAA treatment, the expression of transcription factor genes was upregulated. Then, transcription factors promote the expression of amino acid and ammonium transporter genes. It promotes the expression of other amino acid and nitrogen metabolism related genes, thereby promoting amino acid and nitrogen metabolism.

In our study, the expression levels of most of the studied transcription factors were increased under NAA. This finding suggests that the regulatory role of TFs may play an important role in NAA promoting amino acid and nitrogen metabolism. At present, NAA has been shown to promote the growth of *Armillaria*; however, the molecular mechanism underlying the ability of NAA to promote the growth of *Armillaria* has not been elucidated. Based on this transcriptome analysis and previous studies, a putative regulatory network was proposed whereby NAA stimulated amino acid and nitrogen metabolism to promote *A*. *gallica* growth (Fig 6). Under the NAA treatment, transcription factor gene expression was upregulated, which then regulated the transcription of amino acid and nitrogen metabolism-related genes. These changes may at least partially explain why the biomass of *A*. *gallica* increased under the action of NAA. Further studies should be carried out to better understand the mechanism by which NAA promotes the growth of *A*. *gallica*.

## Conclusion

This study found that 8 mg/L NAA can promote the growth of *A*. *gallica*, and an analysis of the transcriptome sequencing data of *A*. *gallica* identified a total of 2071 DEGs. GO and KEGG pathway enrichment analyses revealed that most of the DEGs were involved in amino acid and nitrogen metabolism under NAA treatment. We also found that the expression levels of genes encoding Gap, AMT, GDH and GS were upregulated. Zn(II)2Cys6 and C2H2 are putative transcription factors related to amino acid and nitrogen metabolism, and they were also identified. This finding revealed that amino acid and nitrogen metabolism-related genes would be rapidly activated by NAA. This study may accelerate the process of revealing the regulatory mechanisms by which NAA promotes the growth of *Armillaria*.

## Supporting information

S1 Fig Expression of glutamine synthetase (GS), glutamate dehydrogenase (GDH) and amino-acid permease inda1 (AAP) genes of *A*. *gallica* after NAA treatment at 2, 4, 5, 6 and 8 h. The y-axis represents the relative gene expression levels analyzed by qRT–PCR. The values are the means ± SE of three biological replicates. Statistically significant differences are indicated by letters above columns (*P* < 0.05, ANOVA).
(DOCX)
