# Supplementary figures and images for: Transcriptome analysis reveals the regulatory mode by which NAA promotes the growth of Armillaria gallica
Source: PLoS One. 2022 Nov 21;17(11):e0277701. doi: 10.1371/journal.pone.0277701 (PMC9678268; doi:10.1371/journal.pone.0277701)

S1 Fig.


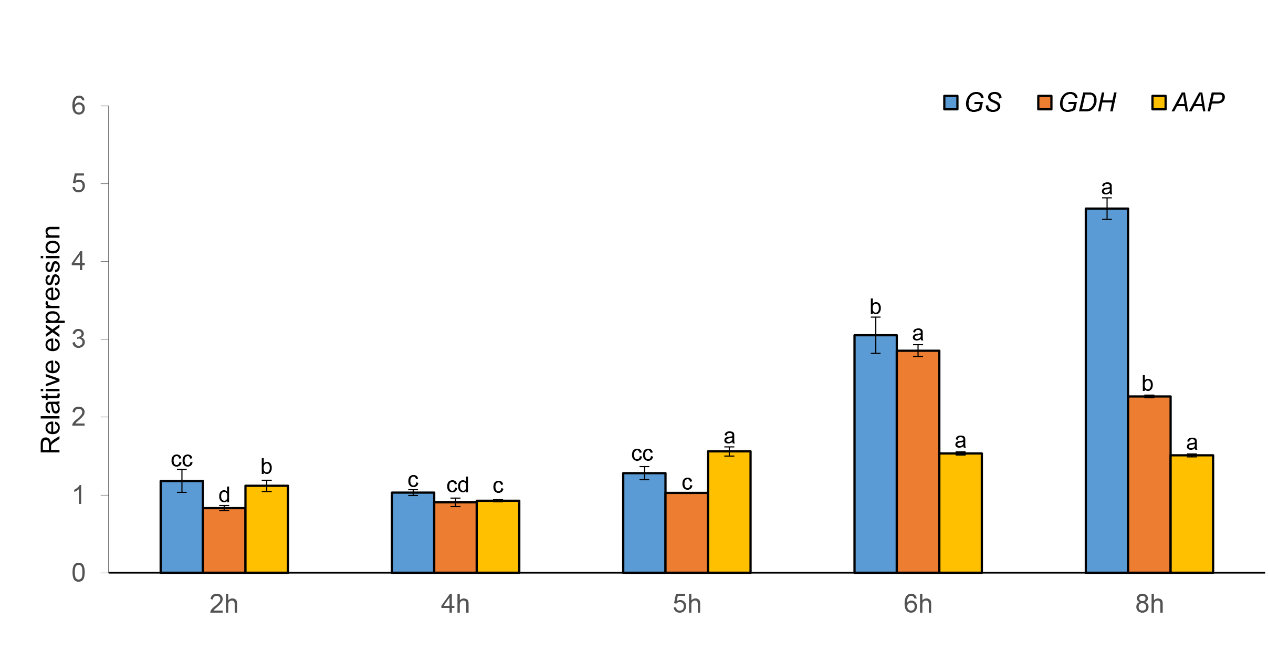

Supplement: S1 Fig — The y-axis represents the relative gene expression levels analyzed by qRT–PCR. The values are the means ± SE of three biological replicates. Statistically significant differences are indicated by letters above columns (P < 0.05, ANOVA). (DOCX) [file pone.0277701.s001.docx]
